# Accumulation of Postoperative Unexpected Events Assessed by the Comprehensive Complication Index^®^ as Prognostic Outcome Parameters for Kasai Procedure

**DOI:** 10.3390/children9101590

**Published:** 2022-10-20

**Authors:** Omid Madadi-Sanjani, Julia Brendel, Marie Uecker, Eva-Doreen Pfister, Ulrich Baumann, Johanna Ohlendorf, Joachim F. Kuebler

**Affiliations:** 1 Department of Pediatric Surgery, Hannover Medical School, 30625 Hannover, Germany; 2 Division of Pediatric Gastroenterology and Hepatology, Department of Pediatric Kidney, Liver and Metabolic Diseases, Hannover Medical School, 30625 Hannover, Germany; 3 Institute of Immunology and Immunotherapy, University of Birmingham, Birmingham B15 2TT, UK

**Keywords:** biliary atresia, unexpected events, clavien-dindo classification, comprehensive complication index

## Abstract

**Introduction** The Kasai procedure in children with biliary atresia (BA) is associated with several complications in the short-term. The Comprehensive Complication Index (CCI^®^) is a validated metric in adult surgery for the analysis of complications and morbidity in surgical patients. We aimed to analyze the CCI^®^ for the first time in BA infants and to correlate its association with outcomes. **Material and Methods** We conducted a retrospective review of medical records of infants with type III BA undergoing the Kasai procedure between January 2011 and December 2021 at our institution. All unexpected events were ranked according to the Clavien–Dindo classification, and the CCI^®^ per patient was subsequently calculated. Clavien–Dindo grades, individual events, CCI^®^, and total event numbers per patient were correlated with one- and two-year outcomes post-surgery. **Results** A total of 131 events were identified in 101 patients (ranging 0–11 per patient). Forty-four Grade I (33.6%), 67 Grade II (51.1%), 18 Grade III (13.7%), and two sentinel events [>Grade IV] (1.5%) were documented according to Clavien–Dindo, including one death in a cardiac-associated BA patient. None of the complications significantly correlated with a poor outcome. Sixty-three (62.4%) CCI^®^ scores were calculated (range 0–100). The mean CCI^®^ score during the in-patient treatment post-surgery was significantly higher in patients with a poorer outcome than patients with native liver survival at one- and two-year follow-up (22.7 ± 21.7 vs. 13.2 ± 18.1; *p* = 0.02). **Conclusion** Not the severity of complications, but the accumulation of numerous events related to Kasai procedure were associated with a poorer outcome. Therefore, the CCI^®^ is an excellent instrument for the postoperative morbidity assessment of BA patients.

## 1. Introduction

Kasai portoenterostomy (KPE) is a palliative surgical procedure in infants with biliary atresia, aiming to restore the biliary drainage and prevent liver transplantation. Though temporary jaundice clearance is achieved in about 50% of the children, liver transplantation is necessary for about 70% of patients in the long-term [1,2,3,4]. A few prognostic parameters for the latter outcome are known at the time of KPE [5,6]. Though disease-related sequelae are common predictors for Kasai outcomes, data on the postoperative complications and morbidity following Kasai procedures are scarce. However, recent studies have shown that surgical and medical errors and events do not affect survival with a native liver in the short- and long-term [7,8]. These studies mainly focused on the analysis of single events in individuals, without putting the adverse events in the context of overall morbidity [9].

The systematic assessment and documentation of postoperative complications in adult hepatobiliary surgery was established early in 2004 with the introduction of the Clavien–Dindo classification system [10]. This instrument focuses on the consequences of complications and grades events according to their severity. 

Recently, the Comprehensive Complication Index (CCI^®^) has been introduced, giving a detailed view of the postoperative morbidity of patients based on adverse events [11,12]. This metric summarizes multiple complications in an individual, based on a prior ranking according to the Clavien–Dindo classification, and we recently reported on its accuracy in pediatric cohorts [13]. Studies in adult oncological cohorts have shown a correlation of the CCI^®^ calculated during the surgical treatment period with long-term outcomes [14,15,16]. For pediatric hepatobiliary diseases, the predictive value of the CCI^®^ has not yet been tested.

The aims of this study were to evaluate the accuracy of the CCI^®^ in the postoperative treatment of BA infants and to correlate its results with short-term outcomes.

## 2. Material and Methods

Ethical approval was obtained from the local ethics committee (Approval no. 9557_BO_K_2021). Informed consent was obtained from legal guardians upon admission.

We conducted a retrospective chart review of patients undergoing Kasai portoenterostomy between January 2011 and December 2021 at Hannover Medical School. For the analysis, all patients with type III biliary atresia, according to the abbreviated Japanese Association of Pediatric Surgeons (JAPS) classification, were included. Patients with syndromic BA, cytomegaly virus positive BA, and other types of BA according to the JAPS classification were excluded from the analysis. The one-year follow-up was available for all (100%) and two-year follow-up data for 86% of the patients through the Biliary Atresia and Related Diseases Registry (www.bard-online.com, accessed on 20 May 2022) [17]. Fourteen patients (14%) did not reach the two-year follow-up mark at the time of the analysis.

The data collection included patient characteristics, age at KPE, liver function tests prior to the KPE, Ishak (semiquantitative fibrosis scoring) interpretation of liver biopsies taken during the KPE, according to a standardized protocol, all unexpected events including surgical and medical interventions (within the first three months following KPE), and Kasai outcome (jaundice-free survival with native liver [<20 μmol/L], survival with native liver, liver transplantation, or death) at the one- and two-year follow-up. 

## 3. Postoperative Kasai Management Protocol

The standardized treatment protocol included the pre- and postoperative administration of ursodeoxycholic acid and fat-soluble vitamins, as well as a 2-week course of intravenous antibiotics (3rd generation cephalosporins or penicillin + beta-lactamase inhibitor) followed by oral prophylaxis for 6 months. The antibiotic drugs were discussed with the Department of Microbiology and were changed based on their recommendations. On the fifth postoperative day, adjuvant treatment with rectal budesonide (2 mg per day) was started and continued for three months [18].

## 4. Definition of Unexpected Events

According to our previously published protocols, unexpected events were defined as any subsequent deviations from the planned perioperative course and changes in the management with any delay in treatment or recovery [13,19,20]. Therefore, we included data on surgical events (redo surgery due to anastomotic leaks, severe bleeding, etc.) and medical interventions not included in our standardized protocol of perioperative care (electrolytes, specific analgetics, albumin substitution, diuretics, transfusions, etc.), taking into consideration that these events are liver disease-related.

## 5. Classification of Unexpected Events

All events were classified according to the Clavien–Dindo system. This severity grading instrument includes five grades, based on the consequences of unexpected events and their severity [10]. Briefly, the classification differentiates between: deviations from the postoperative course without the need for pharmacological treatment or other interventions, with defined allowed therapeutic regimens (Grade I); requiring pharmacological treatment with drugs other than those allowed for Grade I complications (Grade II); interventions not under general anesthesia (Grade IIIa); interventions under general anesthesia (Grade IIIb); single organ dysfunction (Grade IVa); multiorgan dysfunction (Grade IVb); and death of a patient (Grade V). 

We then assessed the CCI^®^ score for all patients, a metric that is based on a prior ranking according to the Clavien–Dindo classification, as an instrument for the postoperative morbidity on a scale of zero (no complication) to 100 (death). For the calculation of the CCI^®^ score, the template available at http://www.cci.assessurgery.com (accessed on 12 June 2022) was used. In this calculation, all events within the follow-up period of 90 days following the Kasai procedure were included.

## 6. Statistical Analysis

Statistical analysis was performed using GraphPad Prism (v8.0; GraphPad Software, San Diego, CA, USA). Spearman’s correlation coefficient was used (*r*) to compare complications and their consequences with the latter outcome; *r*  >  0.8 was defined as strong, and *r* >  0.9 as very strong. Data are presented as mean (s.d.). Statistical significance was set at *p*  <  0.050. Multiple stepwise regression analysis was performed using SPSS version 22.0 (SPSS, Chicago, IL, USA). Each of the unexpected events were analyzed for their association with the one- and two-year outcomes of the BA patients, defined as good (native liver survival and jaundice-free native liver survival) and poor (liver transplantation and death). *p*-values were presented and considered significant with values < 0.05.

## 7. Results

### 7.1. Patient Characteristics

Between the study period of January 2011 and December 2021, 155 infants underwent the Kasai procedure at Hannover Medical School, from which 101 patients (65.2%) matched the inclusion criteria.

The mean age at KPE in the cohort of patients with type III BA was 61 days (±21). The mean liver function tests pre-KPE were 244 U/L (±190) AST, 163 U/L (±118) ALT, 537 U/L (±355) Gamma-GT, and 141 (±45) μmol/L for the conjugated bilirubin.

The native liver survival of patients at the one-year follow-up was 65.3% (66/101) of which 44 (67.0%) were jaundice free; and at the two-year follow-up 54.0% (47/87), with 44 out of 47 (93.6%) being jaundice-free.

### 7.2. Analysis of Unexpected Events

A total of 131 events, according to the Clavien–Dindo classification, in 101 patients were identified (Table 1).

Stratified according to severity, 44 Grade I (44/131; 33.6%) interventions were documented, with the majority being administration of diuretics (n = 39; 88.6%) due to postoperative edema and ascites with an insufficient urine output less than 1.0 cc/h. In three children (6.8%), electrolyte substitutions were necessary (sodium and potassium), and in two children (4.5%), analgetic treatment had to be reintroduced or increased. 

Most events in our cohort were Grade II interventions according to the Clavien–Dindo classification, with 67 in total (67/131; 51.1%). In 30 patients (30/67; 44.8%) early cholangitis was diagnosed with intravenous antibiotic treatment, 10 children (14/67; 14.9%) received parenteral nutrition during hospitalization, and 10 children (14/67; 14.9%) received a transfusion (independent of Kasai procedure). Nine patients (9/67; 13.4%) underwent intravenous antibiotic treatment for central line and wound infections, one (1/67; 1.5%) received antimycotics based on an intra-abdominal swab with signs of a fungal infection and peritonitis, and in eight cases (8/67; 11.9%), albumin substitutions for generalized edema were necessary. 

Two ascites drains (2/131; 1.5%) were placed without general anesthesia (Grade IIIa according to Clavien–Dindo).

Sixteen procedures (16/131; 12.2%) were performed under general anesthesia (Grade IIIb, according to Clavien–Dindo). Five anastomotic leaks (5/16; 31.3%) were documented, one at the portoenterostomy and four at the jejuno-jejunostomy, of which two occurred in the same dystrophic patient. Two re-laparotomies (2/16; 12.5%) were performed due to postoperative intestinal hemorrhage, of which one patient went into cardiopulmonary arrest and resuscitation (Grade IVb), and received a postoperative MRI under general anesthesia (1/16; 6.3%) to rule out cerebral ischemia. Two wound (2/16; 12.5%) and one revision (1/16; 6.3%) of the fascia were performed, and three patients (3/16; 18.8%) received a biloma and another two (2/16; 12.5%) an ascites drain under general anesthesia. 

One Grade V event (death) occurred in a patient with CABA (cardiac-associated biliary atresia) with postoperative cardiac arrest.

### 7.3. Analysis of Unexpected Events and the Kasai Outcome

In the group of 66 patients with native liver survival (SNL) at the one-year follow-up, a mean of 1.1 events (±1.9) occurred within the first three months following KPE, compared with 1.7 (±1.7) in the group of 35 patients with rapid liver deterioration and early liver transplantation (LTx) (*p* = 0.11). Stratified according to the Clavien–Dindo classification, a statistical trend was observed for a higher number of Grade I events in the LTx group (0.6 vs. 0.4; *p* = 0.08), with no statistical difference for the additional Clavien–Dindo grades at one- or two-year follow-up (>*p* = 0.1). 

### 7.4. Comprehensive Complication Index in Our BA Cohort

**Figure 1:**
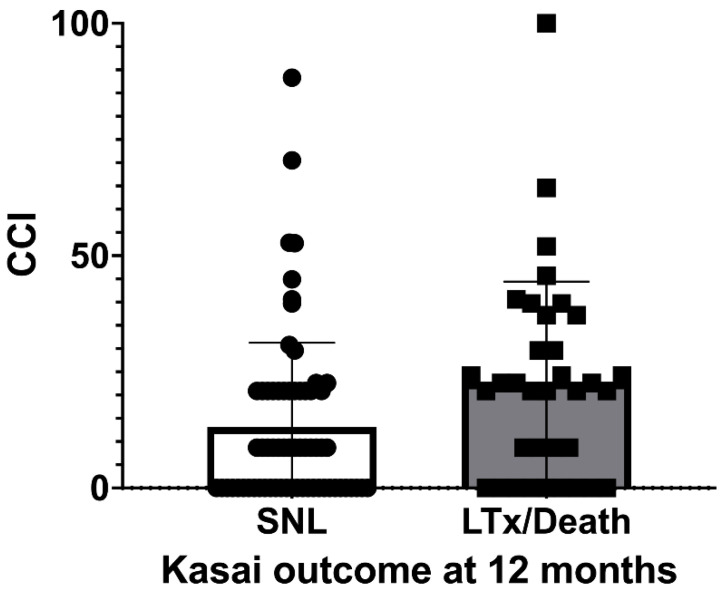
Boxplot showing median CCI^®^ (standard deviations, maximum scores) after Kasai procedure in group of children with native liver survival (SNL) and group with liver transplantation or death (LTx/Death) at 12-month follow-up.

We cumulated multiple events in an individual (within the first three months after KPE) in one CCI^®^. Thirty-eight children (38/101; 37.6%) had no events, and therefore, a CCI^®^ of 0. For 63 BA infants (63/101; 62.4%), a CCI between 8.7 and 100 (death) was calculated. The average CCI^®^ in the cohort was 16.5 (±19.8), with a median of 8.7. A significant difference was detected comparing the SNL group, with a mean CCI^®^ of 13.2 (±18.1), and a median of 8.7, with the LTx/death group, with a mean CCI^®^ of 22.7 (±21.7) and a median of 20.9 (*p* = 0.02) (Figure 1). A significant correlation between the CCI^®^ and outcome at the one-year (*p* = 0.001) and two-year follow-up (0.02) was observed. The CCI^®^ was inversely correlated with the time of transplantation (r = −0.5; *p* = 0.003). Furthermore, the CCI^®^ was significantly correlated (>r = 0.8; *p* = 0.001) with the length of hospital stay (in days) following the Kasai procedure.

In contrast, no significant correlation was detected between the total number of complications per patient (ranging 0–11) and the outcome, without using the severity grading and formula of the CCI^®^ (>*p* = 0.1).

### 7.5. Multivariate Analysis

In a multiple stepwise regression, all complications and their consequences were analyzed for their association with the one- and two-year follow-up outcomes. There was a significant association between the two-year outcome with early postoperative cholangitis (*p* = 0.05) and electrolyte fluctuations and substitutions (*p* = 0.02), whereas a trend was observed for postoperative ascites drains (*p* = 0.1). For re-laparotomies (*p* = 0.4), transfusions (*p* = 0.3), parenteral nutrition (*p* = 0.5), albumin substitutions (*p* = 0.9), and antibiotics for other causes of sepsis (*p* = 0.5), no associations with the outcome were present.

In addition, we did not identify a significant association of early postoperative cholangitis and age at Kasai procedure (*p* = 0.3), preoperative bilirubin levels (*p* = 0.6), and Ishak semiquantitative fibrosis scoring at Kasai procedure (*p* = 0.5).

## 8. Discussion

Kasai portoenterostomy is the first-line treatment for the vast majority of infants with biliary atresia,, achieving biliary drainage in more than 50% of patients [21]. Despite the jaundice clearance, sequelae of portal hypertension are the main drivers for liver deterioration in short- and long-term follow-up [22]. Multimodal treatment strategies, including Kasai procedure and liver transplantation, are necessary in 70–80% of all BA patients, and have continuously increased the overall survival of BA infants in the last decades [23]. However, up to 10% of the children do not survive the transplant waiting lists [24,25]. Therefore, pediatric surgeons and hepatologists have tried to decipher the successful Kasai procedure and a few prognostic markers were identified. The underlying mechanisms of these markers remain cryptic, and only a fraction of BA infants undergoing the Kasai procedure at less than 60 days of age survive with their native liver. 

Calinescu et al. [7] recently discussed whether postoperative complications following the Kasai procedure resulted in rapid liver deterioration and early transplantation. The systematic analysis of postoperative events, according to the Clavien–Dindo classification, did not detect a correlation with the latter outcome. In adult surgery, recent developments of morbidity scores have resulted in applicable prognostic markers for oncological outcomes after surgical interventions. The Comprehensive Complication Index (CCI^®^), a metric from 0 to 100 [death] that includes numerous complications and events in an individual during the in-patient management, is an indicator for postoperative morbidity, and recently, a correlation with the oncological outcomes for colorectal, gastric, and hepatic cancers have been reported [14,26,27].

We therefore translated these concepts into perioperative Kasai management and included all unexpected events, including disease-related interventions (diuretics, electrolytes, albumin etc.) not part of our standardized protocol, and calculated the CCI^®^ for the first time in Kasai surgery. 

Within the study period a total of 131 surgical, non-surgical, and medical interventions were documented, of which two (Clavien–Dindo > Grade IV) were considered sentinel events. There was one death during the in-patient stay, of a child with CABA (cardiac-associated biliary atresia) [28]. For the standardization of our heterogenous cohort, we only included type III BA infants. 

The number of events in our cohort is much a higher compared with other surgical BA collectives in the literature [7,9]. This can be attributed to our definition of unexpected events, including any deviation of the planned postoperative course, that resulted in a delay in treatment and recovery. Therefore, the analysis focused on postoperative morbidity, including KPE-associated events and liver-disease related sequelae. We did not identify any correlation between these events and patient age, degree of liver fibrosis and cirrhosis, and bilirubin and gamma-GT levels at the time of the Kasai procedure. 

As the CCI^®^ is based on a prior ranking of events according to the Clavien–Dindo classification, we first ranked all events according to the Clavien–Dindo instrument and then individually correlated those gradings with the outcome. Though none of the Clavien–Dindo events (Grade I–IV) were significantly associated with a worse outcome at the one- and two-year follow-up, detailed analysis revealed a significant association of electrolyte substitutions (Grade I) for hyponatremia and early postoperative cholangitis (Grade II) with transplantation and death. Hyponatremia is a well-known sequela of pediatric liver diseases, mainly associated with portal hypertension, systemic vasodilatation, and hypovolemia [29]. Low sodium levels are considered a negative predictor for mortality in pediatric liver transplant candidates and, more recently, an association with poor outcome in biliary atresia children with cirrhosis has been reported [29,30]. All children with electrolyte substitutions already had moderate to severe signs of liver cirrhosis in liver biopsies at KPE. 

The entity of postoperative cholangitis in children with biliary atresia is largely unknown, but an ascending bacterial infection from the roux-loop is the main suspect, resulting in long-term antibiotic treatment and prophylaxis [31,32]. A cholangitis incidence between 22 and 79% has been reported in BA infants and recurrent cholangitis episodes are accountable for poorer Kasai outcomes [33,34]. In our cohort, cholangitis incidence was not related to age at Kasai or degree of fibrosis and cirrhosis in the liver biopsy. 

Although a statistical trend was observed for Grade I events (*p* = 0.1) and Kasai outcome, analysis of the CCI^®^ revealed that the cumulation of events mirrored the overall morbidity and as the superior parameter. The mean CCI^®^ in the LTX group was significantly higher compared with the SNL group (22.7 vs. 13.2; *p* = 0.02). Translating CCI^®^ numbers into events per patient showed that children in the SNL, on average, suffered one to two Grade I events (Grade I = CCI^®^ equivalent 8.7), whereas the LTx group experienced one and more Grade II events (Grade II = CCI^®^ equivalent 20.9). The combination of events within these groups were inconsistent and the multivariate analysis did not identify individual events to be significantly associated with the outcome. Furthermore, the CCI^®^ data confirmed the results of previous publications on surgical (interventional) complications and events (>Grade IIIa = CCI^®^ equivalent > 26.2) that did not present an association with the native liver survival. 

Though Grade IIIa events and higher are significant drivers of overall postoperative outcome using the CCI^®^ [15,26] in adult oncological surgery, our study confirmed that variations in the postoperative pharmacological treatment were important predictors for the outcome of Kasai procedure. 

In addition to our previous report on the feasibility of the CCI^®^ in a general pediatric surgical cohort, the authors in Fuchs et al. more recently reported on the utilization of the CCI^®^ in pediatric liver surgery [13,35]. Not only did the authors report on the advantages of the CCI^®^ for the standardization of outcome measures, they also emphasized its advantages in establishing standardized measures of quality in pediatric surgery. Experiences in adult surgery already show that the CCI^®^ can be evaluated for comparison between surgical techniques and for comparison of perioperative morbidity and mortality between institutions. Therefore, the CCI^®^ should be considered a potential indicator for surgical quality in pediatric surgery. 

Although further validation of the CCI^®^ in Kasai procedure is necessary, we recommend its usage for future studies in BA surgery.

## 9. Limitations

Several limitations of our study need to be addressed. The medical records were retrospectively analyzed, which may have led to missing values in our cohort. Furthermore, the events were retrospectively ranked according to the Clavien–Dindo classification and the CCI^®^, and a plausibility validation of the historical data was not possible in all cases. Finally, the indication for pharmacological treatment underwent protocol changes during the study period, which may have led to a lower threshold for the cholangitis and diuretic treatments in some patients.

## Figures and Tables

**Table 1:** Information on all unexpected events in the postoperative management of biliary atresia following Kasai procedure and grading according to the Clavien–Dindo classification.

Clavien-Dindo Grade	Type	n=	Total (n)
**I**	Diuretics Analgetics Electrolytes	39 2 3	**44**
**II**	Parenteral nutrition Transfusion Antibiotics for wound infection Antibiotics for sepsis Antimycotics for intra-abdominal fungal infection Antibiotics for cholangitis Albumin substitution Vitamin K IV substitution	10 10 1 8 1 30 6 1	**67**
**IIIa**	Ascites drain	2	**2**
**IIIb**	Wound revision Re-laparotomy for bleeding Ascites drain Biloma drain Fascia dehiscence Anastomotic leak MRI to rule out cerebral ischemia	2 2 2 3 1 5 1	**16**
**IVa**	/	0	**0**
**IVb**	Cardiopulmonary resuscitation	1	**1**
**V**	Cardiac arrest	1	**1**

## Data Availability

Not applicable.
